# Plasmatic and cell-based enhancement by microparticles originated from platelets and endothelial cells under simulated in vitro conditions of a dilutional coagulopathy

**DOI:** 10.1186/s13049-021-00847-9

**Published:** 2021-02-23

**Authors:** Julia K. Böhm, Nadine Schäfer, Marc Maegele, Birgit Stümpges, Ursula Bauerfeind, Michael Caspers

**Affiliations:** 1 The Institute for Research in Operative Medicine, Faculty of Health, Department of Medicine, Witten/Herdecke University, Ostmerheimer Straße 200, 51109 Cologne, Germany; 2 Department of Traumatology, Orthopaedic Surgery and Sports Traumatology, Cologne-Merheim Medical Centre (CMMC), Witten/Herdecke University, Campus Cologne-Merheim, Ostmerheimer Str. 200, D-51109 Cologne, Germany; 3 Department of Haematology and Transfusion Medicine, Cologne-Merheim Medical Centre (CMMC), Witten/Herdecke University, Campus Cologne-Merheim, Ostmerheimer Str. 200, D-51109 Cologne, Germany

**Keywords:** Lethal triad, Platelet-derived microparticles, Endothelial-derived microparticles, Trauma-induced coagulopathy

## Abstract

**Background:**

Aggressive fluid management and other external factors may lead to hypothermia, acidosis and hemodilution (defined as Lethal Triad, LT) contributing to a trauma-induced coagulopathy (TIC) that worsens patients’ outcomes. Procoagulant microparticles (MP) are crucial players at the interface of cellular and plasmatic coagulation. However, their functions remain largely unexplored. This study aimed to characterize effects of MP subtypes and concentrations on functional coagulation under in vitro simulated conditions.

**Methods:**

Blood from eleven volunteers were collected to simulate in vitro conditions of hemodilution (HD) and LT, respectively. HD was induced by replacing a blood volume of 33% by crystalloids and for LT, samples were further processed by reducing the temperature to 32 °C and lowering the pH to 6.8. MP were obtained either from platelet concentrates (platelet-derived MP, PDMP) or from cell culture (ECV304 cells for endothelial-derived MP, EDMP) by targeted stimulation. After introducing MP to in vitro conditions, we measured their concentration-dependent effects (1.000, 10.000 and 15.000 MP/μl blood) on coagulation compared to whole blood (WB). For each condition, coagulation was characterized by flow cytometric platelet activation and by quantification of fibrin clot propagation using Thrombodynamics® technology.

**Results:**

MP originated from platelets and endothelial cells affected blood coagulation in a concentration-dependent manner. Particularly, high PDMP quantities (10.000 and 15.000 PDMP/μl blood) significantly induced platelet activation and fibrin clot growth and size in HD conditions. In LT conditions as well, only high PDMP concentration induced platelet activation, clot growth and size. In contrast, EDMP did not induce platelet activation, but resulted in enhanced formation of spontaneous clots, irrespective of simulated condition. With increasing EDMP concentration, the time until the onset of spontaneous clotting decreased in both HD and LT conditions.

**Discussion:**

The study demonstrates an essential role of MP within the coagulation process under simulated coagulopathic conditions. PDMP affected platelets promoting clot formation likely by providing a surface enlargement. EDMP presumably affected clotting factors of the plasmatic coagulation resulting in an increased formation of spontaneous clots.

**Conclusion:**

Under simulated conditions of a dilutional coagulopathy, MP from different cellular origin indicate a divergent but both procoagulant mechanism within the coagulation process.

## Introduction

Despite continuous improvements in trauma management, traumatic injuries are still the leading cause of death and disability in adults under 40 years [1–3]. Particularly, uncontrolled bleeding contributes to more than 50% of all trauma-related deaths. The bleeding phenotype is significantly aggravated by a trauma-induced coagulopathy (TIC) occurring within hours after injury [4, 5]. Approximately, one of four trauma patients arrive at the emergency department with laboratory signs of a compromised coagulation resulting in a four-fold higher mortality [6–8]. Efforts to elucidate the underlying pathomechanism led to improved resuscitation strategies in trauma management over the last decade, but still remain unknown in decisive parts [9, 10]. Looking at etiology, the described mechanisms are currently divided into either a trauma and/or traumatic shock-induced endogenous coagulopathy (including endotheliopathy) also described as acute traumatic coagulopathy (ATC) or iatrogenic coagulopathy (IC) [3, 11, 12]. IC is triggered by an aggressive trauma and in particular fluid management that may lead to hypothermia, acidosis and hemodilution, which worsens the outcomes of severely injured patients significantly [12]. Due to the high impact on coagulation of these three external conditions, they are most recently referred as “lethal triad” (LT) although hemodilution was not part of the historical definition. A progressive course of LT is associated with a deterioration of coagulation accompanied by disturbed clot formation and strength [13–15]. For primary hemostasis and particularly platelet function under clinical conditions of ATC, a significant decrease in platelet activation and aggregation leading to deterioration of clot formation was described [16, 17]. But in contrast to this mechanism, functioning at this boundary of cellular- and plasmatic-driven coagulation, small cell-derived subcellular vesicles defined by size of 0.1–0.9 μm (known as microparticles, MP) are produced and released in large quantities from different cell types [18–21]. MP therefore are mediators of the cellular and plasmatic coagulation and interact with various systemic inflammatory pathways initiated after trauma and other acute-phase conditions (e.g. ARDS, [22] and multiple organ failure [21, 23]). There is evidence that different MP phenotypes trace back their cellular origin depending on their membrane antigen composition. In addition, after major trauma, a characteristic distribution pattern correlating with injury severity could be shown [24, 25]. This study aimed to characterize effects of MP subtypes and concentrations on functional coagulation under in vitro simulated coagulopathic conditions. In relation to their potential to facilitate coagulation complexes and initiate the coagulation process via TF−/FVII-dependent and independent pathways, our goal was to understand and to differentiate the role of platelet- and endothelial-derived MP (PDMP, EDMP) on functional coagulation [26, 27]. Increasing MP concentrations were used to investigate their impact on primary and plasmatic hemostasis under simulated standardized in vitro IC-conditions.

## Methods

### *Targeted* in vitro synthesis of microparticles

#### Cell line and cultivation for EDMP production

Human ECV304 cells (Sigma-Aldrich, Steinheim, Germany) were used to investigate endothelial MP generation properties. Cultivated in Dulbecco’s Modified Eagle Medium (DMEM) and incubated under constant conditions at 37 °C, 5% CO_2_ and 90% humidity, cells were subcultured by trypsinization (0.25% trypsin, Sigma-Aldrich, Steinheim, Germany).

For generating particles, ECV304 cells were incubated with 1 mM hydrogen peroxide (Sigma-Aldrich, Steinheim, Germany); platelets with 1.5 μg/ml bacterial lipopolysaccharide (LPS) (Sigma-Aldrich, Steinheim, Germany) – both for 22 h at 37 °C maintaining established cultivation conditions. Subsequently, a first centrifugation step was performed to remove cells (10 min at 1.000 x g, 4 °C) followed by a second step to sediment particles from transferred supernatant (45 min at 10.000 x g, 4 °C). After removing residual supernatant, pellets were resuspended in 1x phosphate buffered saline (PBS), pooled and stored at − 80 °C until analysis.

### Platelet concentrates and PDMP production

Human platelets were obtained from platelet apheresis concentrates of the Institute of Transfusion Medicine (ITM) of the Cologne-Merheim Medical Centre that were not being authorised for transfusion.

For in vitro synthesis of PDMP, 40 mls of platelet concentrates were stimulated with 1.5 μg/ml LPS for 22 h at 37 °C in a humidified incubator with 5% CO_2_ (ThermoFisher, Marietta, USA). Subsequently, the platelet suspension was centrifuged at 1.000×g for 10 min at 4 °C (Heraeus, Hanau, Germany). Cell-free supernatant was centrifuged twice at 10.000×g for 45 min at 4 °C (Heraeus, Hanau, Germany) for PDMP pellet sedimentation, which was resuspended in the following in PBS obtaining pure PDMP. PDMP pellets were pooled, aliquoted and stored at − 80 °C until analysis.

### Blood donation and processing for inducing coagulopathic conditions

Study approval was given by the ethical committee of Witten/Herdecke University (#182/2016). Eleven healthy volunteers, fulfilling the ITM criteria for blood donation (age ≥ 18 years; no preexisting history of coagulation disorders, anticoagulant and/or platelet-inhibiting medication or viral infection), consented to participate and donated 60 mls blood, which was collected in citrated monovettes (Sarstedt, Nümbrecht, Germany).

Coagulopathic conditions such as haemodilution (HD) and lethal triad (LT) were simulated in vitro as previously described [28, 29]. In brief, conditions were either induced by diluting whole blood (WB) by replacing 1/3 by crystalloids (Sterofundin® ISO Infusionslösung, B. Braun Melsungen AG, Melsungen, Germany) only (hereafter be referred to as haemodilution, HD) or in combination with lowering the pH value to 6.8 using 2 M HCl and decreasing the temperature from 37 to 32 °C (hereafter be referred to as lethal triad, LT). Constant temperature was realized by continuous storage of samples in an appropriate tempered water bath (Thermolab1070, GFL, Burgwedel, Germany).

### Application of PDMP and EDMP to the simulated HD and LT approaches

**Fig. 1:**
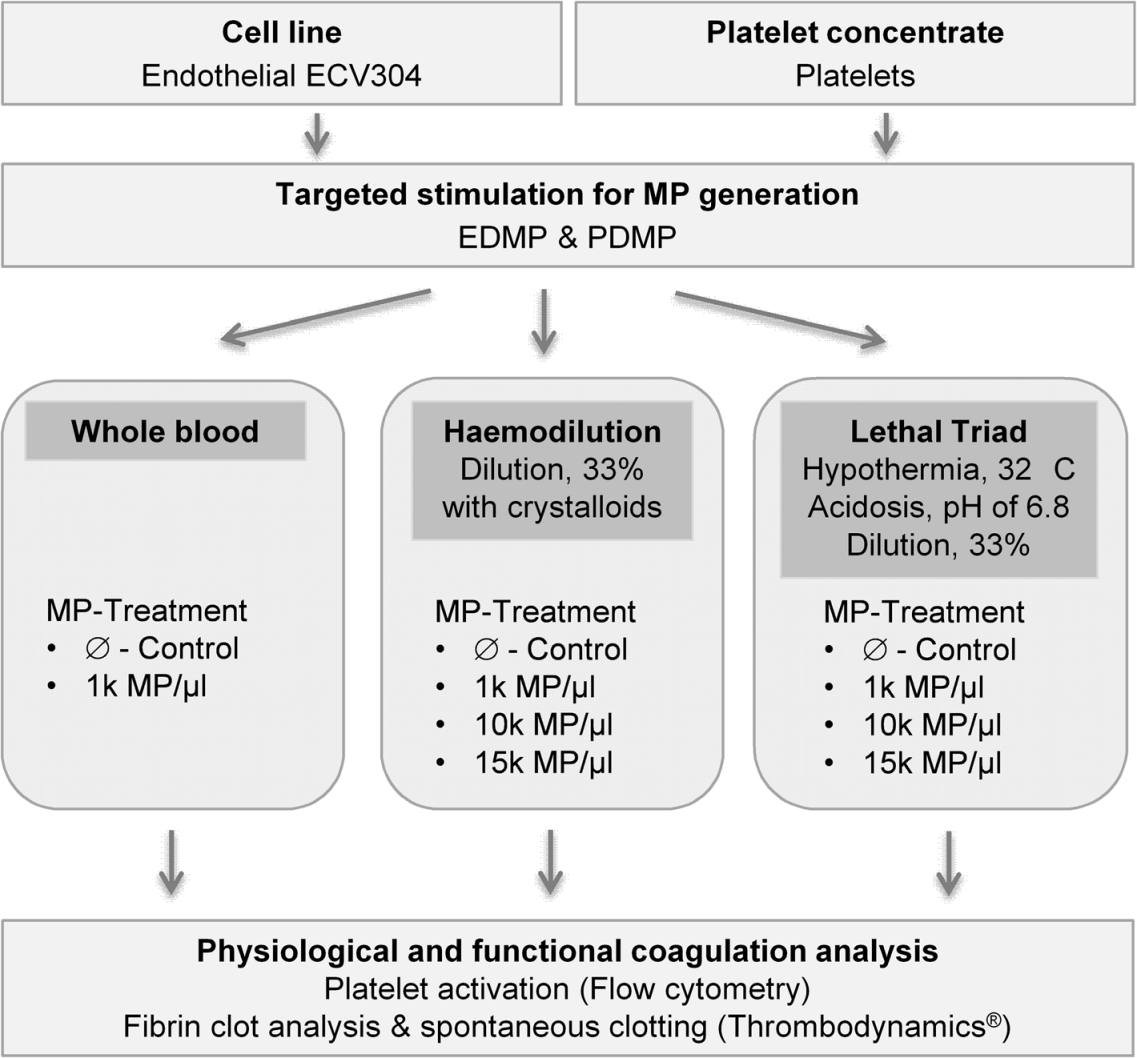
Schematic representation of experimental design

After introducing the conditions of HD and LT, either no MP (untreated controls) or EDMP/PDMP were supplemented in distinct concentrations of 1000 (1 k), 10.000 (10 k) or 15.000 (15 k) MP/μl to the respective condition or whole blood (Fig. 1). Exact microparticle quantities required for the simulated approaches had been determined shortly before flow cytometric analysis by using the BD Accuri™ C6 (BD, Heidelberg, Germany). For this purpose, particles were defined by size (0.5 to 0.9 μm) [21] and by typical surface marker originated from the parental cell such as CD42b for platelets and CD144 for endothelial cells, respectively (BD, Heidelberg, Germany). In addition, Annexin V dye was used as marker for the externalisation of phosphatidylserine (PS). After a microparticle incubation of 5 min, samples were processed for subsequent coagulation analysis.

### Extended coagulation analysis

#### Detection of activated platelets

For measuring platelet activation, cells were fixed according to Cyfix III protocol [30] and P-selectin expression was flow cytometric measured by using CD42b and CD62p antibodies (BD. Heidelberg, Germany) and the BD Accuri™ C6 device (BD, Heidelberg, Germany). Results are presented as relative changes of CD42b^+^/CD62p^+^ stained platelets referring to the respective unstimulated approach of each experimental group (set as 100%).

### Thrombodynamics® (TD)

Differential centrifugation was applied for TD analysis (1.600 x g for 15 min followed by a spin of plasma for 5 min at 10.000 x g) and platelet-poor plasma (PPP) was immediately shock-frozen in liquid nitrogen and stored at − 80 °C until analysis.

For TD analysis, PPP samples of WB and HD were thawed at 37 °C and those of LT were thawed accordingly at 32 °C in a water bath. The corresponding temperatures for WB, HD and LT were maintained during the TD measurements.

For the determination of spatial clot growth, the TDX kit consisting of reagent I (lyophilized protein of FXIIa inhibitor) and reagent II (CaCl_2_) was used according to the manufacturer’s recommendation (HemaCore, Moscow, Russia). Briefly, 120 μl of PPP were supplemented with reagent I and incubated in the device thermostat for 15 min (HemaCore, Moscow, Russia). Subsequently, the PPP samples were treated with reagent II and directly placed into the micro chamber. By inserting immobilized tissue factor, the reaction had been initiated and clot growth and spontaneous clot formation was recorded over 45 min. The following parameters were measured: lag-time (Tlag, min), rate of clot growth (V, μm/min), initial rate of clot growth (Vi, μm/min), clot density (D, a.u.) and clot size (Cs, μm). In addition to these parameters, Cs was measured in five minutes intervals (0–20 min) to estimate the influence of MP on the dynamics of clot growth. In order to statistically record the changed Cs over time, the individual areas under the curve (AUC) of each donor was calculated.

### Statistical analysis

Statistics and graphical data analyses were performed using GraphPad Prism version 7.00 for Windows (GraphPad Software, La Jolla, USA). Non-parametric Friedman test and Dunn’s post-hoc test to correct for multiple comparisons were applied to determine significant differences across the collected parameters in the groups of HD and LT.

In a first step, the MP-untreated samples in the groups of WB, HD and LT were compared in pairs as follows: WB vs. HD, WB vs. LT and HD vs. LT. Then, the effects of dose-specific application of PDMP or EDMP were tested in the HD and LT groups by comparing those two. Finally, using a non-parametric Wilcoxon test potential effects between untreated and MP-supplemented samples (C vs. 1 k) were analyzed. *P*-values with a significance level < 0.05 were considered statistically significant.

Values with respect to platelet count and TD temporal course of clot growth (Cs) are presented as arithmetical mean and standard deviation.

## Results

### Demographics of healthy volunteers

Overall, 11 healthy donors were enrolled for analysis. 54.5% of donors were males with a median age of 42 years [IQR 22].

### Effect of simulated conditions and MP supplementation on platelet count and activation

**Fig. 2:**
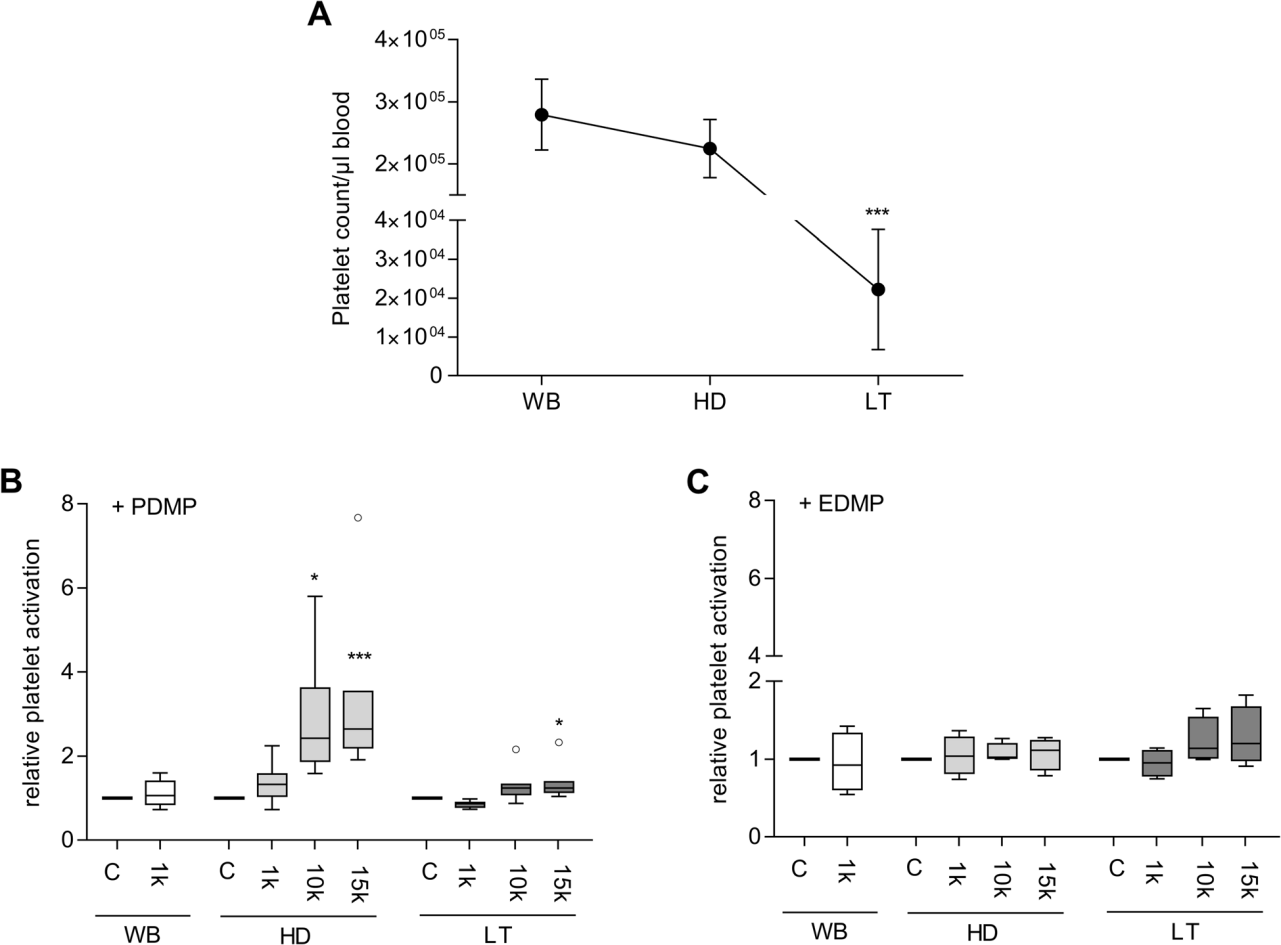
Platelet count (**a**) and platelet activation after concentration dependent PDMP (*n* = 7) (**b**) and EDMP (*n* = 4) (**c**) supplementation under the simulated conditions of whole blood (WB), haemodilution (HD) and Lethal Triad (LT). Platelet activation was measured by expression levels of CD42b^+^/CD62p^+^ on platelets surface. Results are presented as relative changes referring to the respective unstimulated approach of each experimental group. Values are presented as mean/median ± SD (**a**) or as Tukey boxplots with whiskers of length 1.5 x IQR (**b**, **c**). Statistical significances were marked with asterisks for concentration-dependent differences within one experimental group (*^,+^*p* ≤ 0.05, **^,++^*p* ≤ 0.01, ***^,+++^*p* ≤ 0.001), respectively. The abbreviation k signifies a thousand

While the condition of HD led to a marginally decreased platelet count than WB (WB: 2.7 × 10^5^/μl, [IQR: 0.7 × 10^5^/μl] vs. HD: 2.2 × 10^5^/μl, [IQR 0.6 × 10^5^/μl]), the LT caused a significant reduction of cell numbers compared to WB samples (LT: 2.1 × 10^4^, [IQR 2.2 × 10^3^/μl]; *p* ≤ 0.001; Fig. 2 a).

The application of MP resulted in a varying platelet activation depending on the supplemented concentration. Under HD conditions, high PDMP quantities (10 k and 15 k MP/μl blood) induced an activation of platelets, which was demonstrated by a significant increase of the cell surface marker CD42b^+^/CD62p^+^ expression compared to controls (Fig. 2 b). Particularly the administration of 15 k PDMP/μl blood further increased platelet activity in this group. Similarly, the highest PDMP concentration (15 k MP/μl blood) induced the greatest effect on platelet activation within the LT group compared to the untreated LT controls (Fig. 2 b). In contrast to PDMP, EDMP supplementation did not affect platelet activation significantly either group. However, a trend of slight enhancement of activated platelets was observed after supplementation of 10 k and 15 k EDMP/μl blood (Fig. 2 c). Irrespective of the supplemented MP subtype, low concentration (1 k of any MP/μl blood) had no effect on platelet activation (Fig. 2 b, c).

### Kinetics of fibrin clot formation and density after PDMP application

The kinetics of clot formation measured by Tlag, clot growth (V) and the initial rate of clot growth (Vi) remained unaffected in the experimental controls of WB, HD and LT. Only after introduction of low concentrated PDMP (1 k PDMP/μl blood) to the whole blood condition a significant change of all clot formation parameters could be observed but remained unaffected under simulated conditions of HD and LT (Table 1). In HD, high PDMP concentrations (10 k and 15 k PDMP/μl blood) resulted in an enhancement of both growth parameters (Table 1). Under LT conditions, the application of 10 k and 15 k PDMP/μl blood resulted in an even stronger increase of fibrin growth rate (V) compared to untreated LT controls (Table 1). Furthermore, a tendential but not significant increase of initial clot growth could be observed after PDMP administration in LT samples (Table 1).

**Table 1:** Kinetics of fibrin clot formation and density after PDMP application

Parameters	PDMP									
	WB		HD				LT			
	C	1 k	C	1 k	10 k	15 k	C	1 k	10 k	15 k
Tlag [min]	0,8 (0,2)	1* (0,2)	0,7 (0,1)	0,8 (0,3)	0,8 (0,2)	0,9* (0,1)	0,9 (0,1)	0,9 (0,2)	0,9 (0,1)	0,9 (0,1)
V [μm/min]	25,5 (5,2)	30,8^*^ (3,5)	28,5 (5,7)	30,5 (3,5)	38^**^ (5,1)	39,8^***^ (3,2)	44,4^+++^ (6,2)	46 (11,4)	49,3^*^ (16,2)	54,25^**^ (11,37)
Vi [μm/min]	54,2 (8,7)	59,4* (9)	55,7 (9,6)	61,8 (5,7)	71,6*** (2,6)	69** (3,8)	69,5^++^ (4,1)	70,4 (6,1)	70,9 (5)	71,6 (6,2)
D [a.u]	19.209 (5.878)	21.637^*^ (6.089)	16.570 (4.876)	17.284 (6.098)	16.611^*^ (5.469)	17.097^*^ (6.864)	14.797^+++^ (4.586)	14.690 (2.951)	14.567 (3.625)	15.488 (3.447)

The following kinetic parameters were determined: Tlag: initial growth rate, V: average clot growth rate and Vi: initial clot growth rate. Clot density was measured as dynamic parameter. Values are presented as median with the corresponding IQR. Statistical significances were marked with plus symbols for differences between unstimulated groups (WB vs. HD and WB vs. LT) or with asterisks for concentration-dependent differences within one experimental group (*^,+^*p* ≤ 0.05, **^,++^*p* ≤ 0.01, ***^,+++^*p* ≤ 0.001), respectively. The abbreviation k signifies a thousand

In contrast to WB, fibrin polymerization and the associated clot density (D) deteriorated under simulated in vitro HD and LT conditions (Table 1) while a significant reduction compared with untreated WB was detected for LT (Table 1). An improved clot density of fibrin polymerization was achieved by PDMP quantities of 1 k MP/μl blood in WB and by high PDMP quantities (10 k and 15 k MP/μl blood) in HD samples (Table 1). A PDMP stimulating effect on clot density could not be detected for LT (Table 1).

### Fibrin clot formation following PDMP supplementation - growth and size

**Fig. 3:**
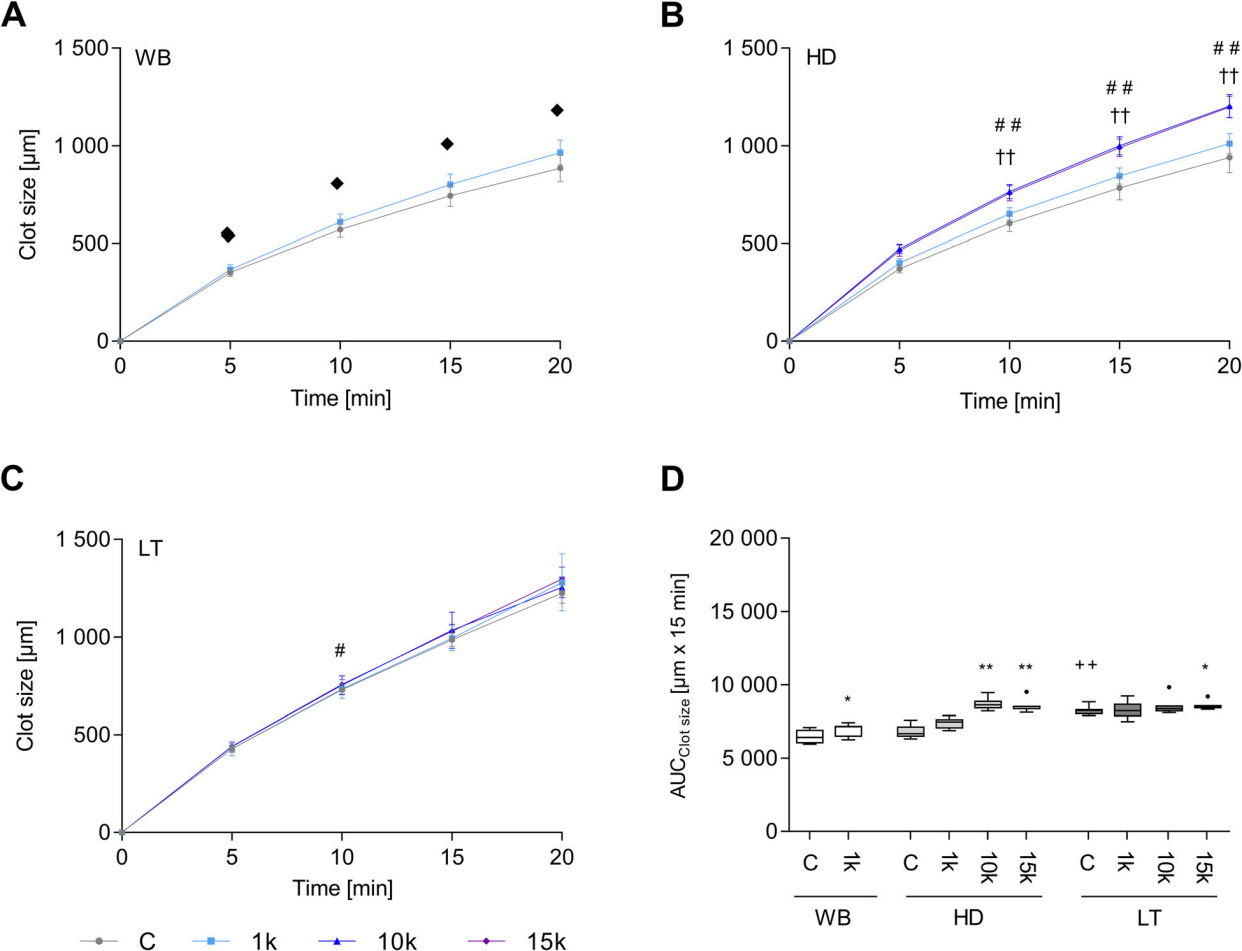
Dynamic fibrin increases over time measured by clot size in whole blood (WB), haemodilution (HD) and lethal triad (LT) after PDMP supplementation (**a**-**c**). Values are represented as mean ± standard deviation. The respective AUCs at 15 min (**d**) following measurement initiation are depicted as Tukey-boxes. Significances are indicated with the following symbol for the following comparisons: diamond - controls vs. 1 k PDMP, cross – controls vs. 10 k PDMP and hash - controls and 15 k PDMP. Statistical significances regarding AUCs were marked with plus symbols for differences between unstimulated groups (WB vs. HD and WB vs. LT) or with asterisks for concentration-dependent differences within one experimental group. Irrespective the comparison one symbol indicates p ≤ 0.05, two symbols p ≤ 0.01 and three symbols p ≤ 0.001). The abbreviation k signifies a thousand

**Fig. 4:**
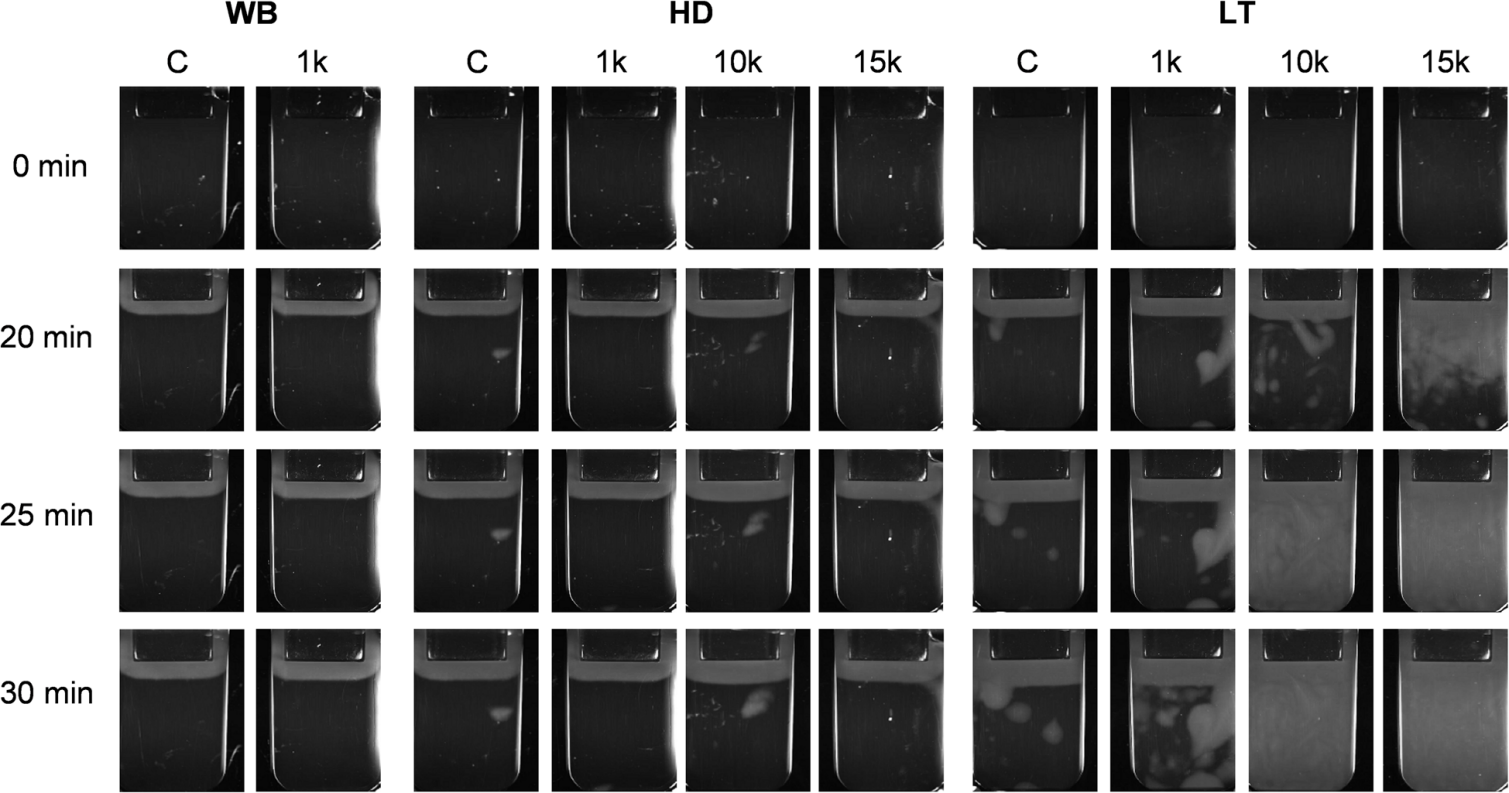
Fibrin growth and development over time after PDMP supplementation in increasing concentrations (1 k, 10 k and 15 k) in whole blood (WB), haemodilution (HD) and lethal triad (LT) groups

The concentration dependent PDMP application resulted in a significantly increased fibrin clot growth (Cs) over the measured time of 20 min in the WB and HD groups (Fig. 3). While the administration of low PDMP concentration 1 k PDMP/μl blood caused an enhanced clot growth in WB (Fig. 3 a), similar effects had been achieved in HD after supplementing high level of PDMP (10 k and 15 k PDMP/μl blood, Fig. 3 b and Fig. 4). The improved fibrin formation in WB and HD was also mirrored in the respective AUCs over a time course of 15 min (Fig. 3 d).

Noteworthy, the condition of LT alone led to an increased fibrin growth 15 min after measurement initiation compared to WB controls (Fig. 3 d). However, the supply of PDMP had no further impact except for the addition of 15 k PDMP/μl blood at the time point of 10 min (Fig. 3 c).

### Spontaneous clotting after EDMP supplementation

**Fig. 5:**
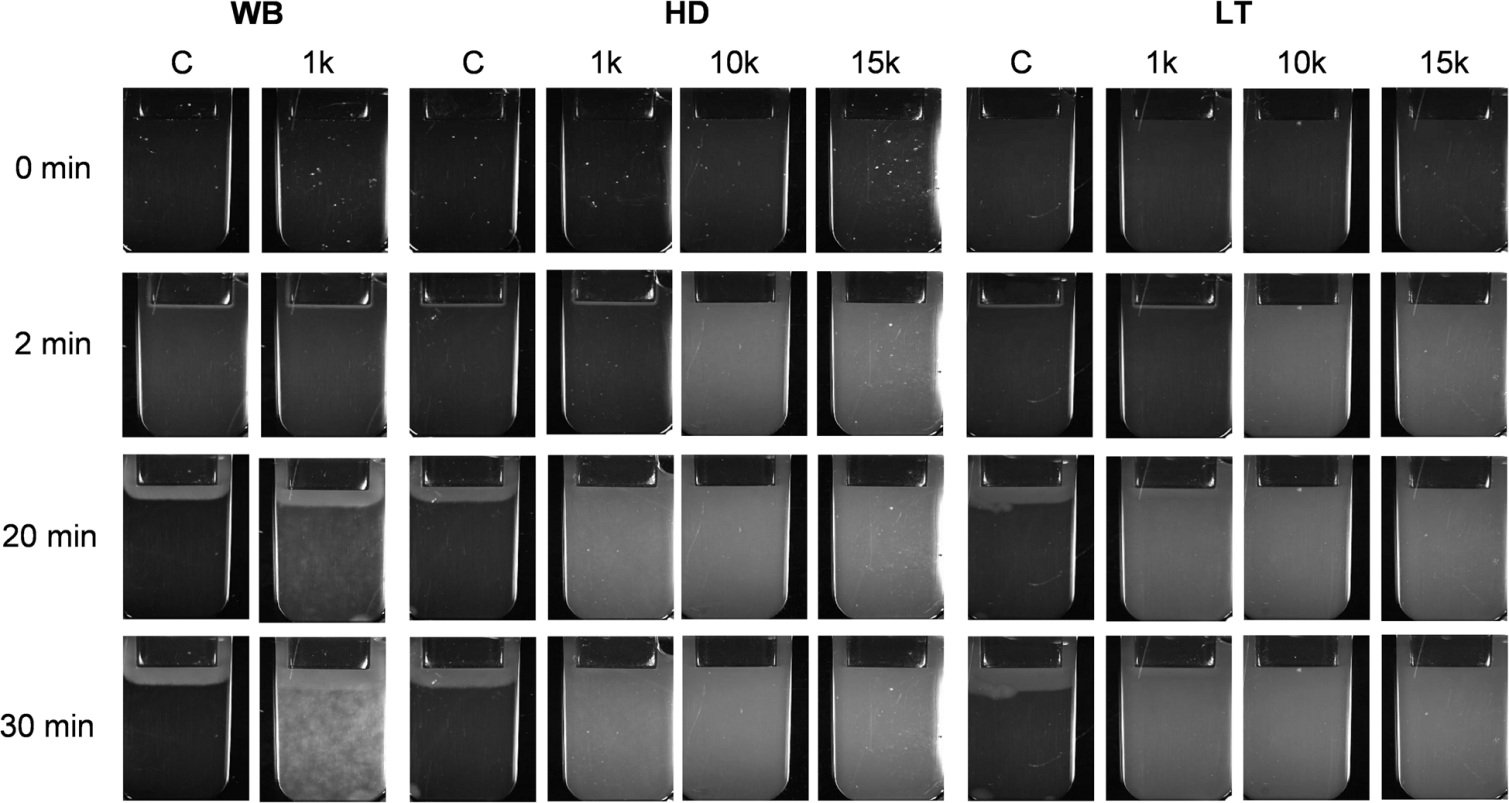
Fibrin growth and development over time after EDMP addition in increasing concentrations (1 k, 10 k and 15 k) in whole blood (WB), haemodilution (HD) and lethal triad (LT) groups

**Fig. 6:**
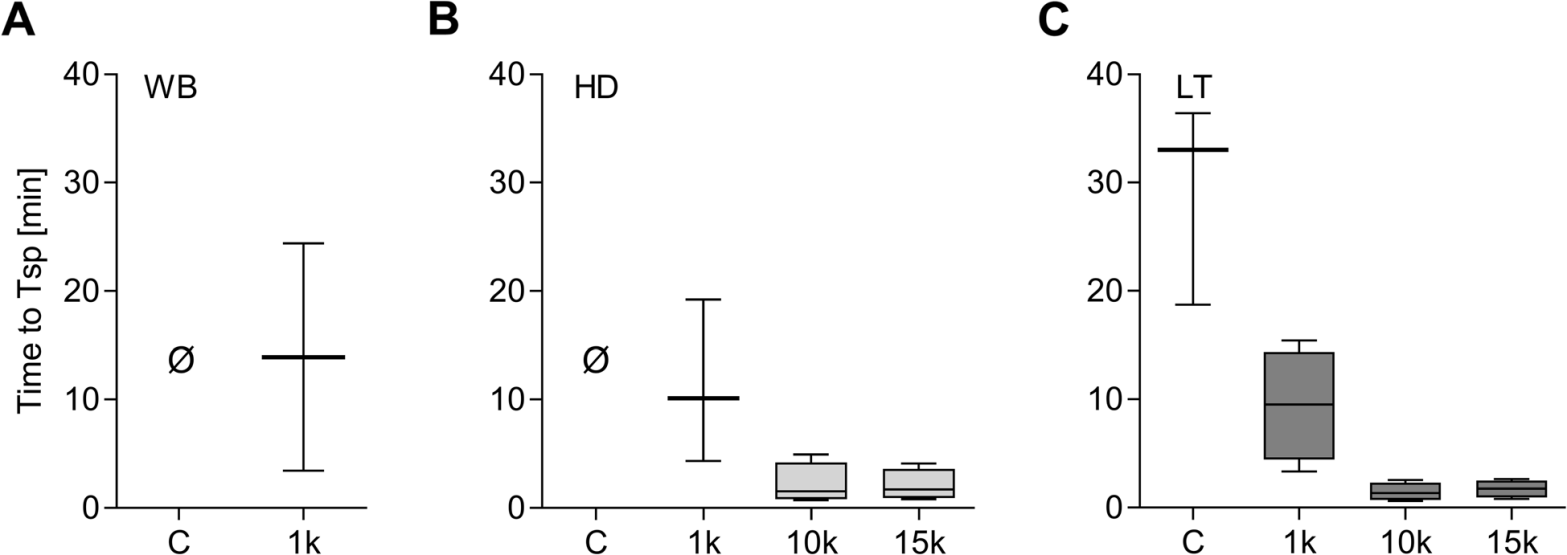
The time elapsed until spontaneous clotting (Tsp) after increasing EDMP supplementation under simulated condition of whole blood (WB), haemodilution (HD) and lethal triad (LT). The abbreviations k signifies a thousand and Ø no occurrence of spontaneous clotting

Data collection for the parameters defining fibrin clot growth could not be collected in the EDMP groups due to the early formation of spontaneous clotting originating from EDMP that occurred irrespective of the simulated setting (Figs. 5, 6). While no spontaneous clot formation was observed in the control groups of WB and HD, it did occurr in LT control conditions (Fig. 6).

Even low EDMP concentrations (1 k EDMP/μl blood) caused a median spontaneous clot formation in WB (13.4 min, [IQR 21 min]), HD (10.1 min, [IQR 14.9 min]) and LT samples (9.5 min, [IQR 9.6 min]) and resulted in unrestricted formation of fibrin clots (Figs. 5, 6 a-c). While the supplementation of high EDMP quantities (10 k and 15 k EDMP/μl blood) initiated spontaneous clotting in HD samples after one minute (1.55 min, [IQR 3.4 min] vs. 1.7 min, [IQR 2.7 min]; Fig. 6 b), a firm clot filling in the whole measuring cuvette was observed after two minutes (Fig. 5). Within the LT condition a similar effect could be observed when supplementing high EDMP concentrations (10 k EDMP/μl blood: 1.35 min, [IQR 1.6 min]; 15 k EDMP/μl blood: 1.75 min, [IQR 1.45 min]; Fig. 6 c and Fig. 5). In all simulated approaches, spontaneous clot formation led to the expansion of clot propagation up to a firm clot in the measuring cuvette, which originated from the spontaneous clotting spots.

## Discussion

The present study aimed to elucidate the role of PDMP and EDMP on functional coagulation under in vitro simulated conditions. In line with previous findings, the applied experimental settings of HD and LT in this study were appropriate models for standardized in vitro simulations of an dilutional coagulopathy that may occur after trauma [28, 29].

With this study we demonstrated for the first time a divergent mechanism of action on coagulation originating from MP with different parental cells. Special emphasis was given to the analysis of selected MP on blood coagulation with a particular focus on PDMP and EDMP. Both MP subtypes had been reported to be released in high quantities and a specific distribution pattern in patients who sustained major traumatic injuries. But whether these particles contribute to the pathomechanism of TIC or are involved in a regulation pathway after trauma is still a matter of research [19, 24, 25, 31]. Within our study design, supplemented PDMP mainly affected circulating platelets by inducing their activation, which was measured by enhanced P-selectin expression on platelet membranes (CD42b^+^/CD62p^+^), particularly, but not exclusively, in HD conditions. It is conceivable that an increased platelet activation likely compensates for reduced platelet counts in HD and LT conditions potentially via the ligand-receptor interaction between the P-selectin and P-selectin glycoprotein ligand-1 (PSGL-1) forming a PSGL-1/P-selectin complex that leads to exponential cell activation [32–35]. In trauma patients it has been demonstrated that increased cell activation is associated with a promoted phosphatidylserine (PS) exposure resulting in procoagulant platelet formation and increased procoagulant activity of PS positive MPs [18, 19, 36]. In addition, this activation lead to an release of coagulation-promoting components from the platelet storage granules [37]. Furthermore, not only platelets expose PS on their membrane, but also in vitro generated PDMP as determined in this study by flow cytometric measurements. It is well known that PS on platelet surfaces cause an assembly of coagulation factors promoting functional clotting [38, 39]. Related to our findings Lipets et al. also revealed that in vitro generated PDMP contribute to an enhanced coagulation propagation by expressing PS on their cell membrane [40].

PDMP together with activated platelets could result in a surface expansion for clotting factors, which then would explain the locally increased formation of thrombin and fibrin polymerization under high PDMP treatment mainly in HD samples.

In contrast to PDMP, in vitro generated EDMP affected the impaired coagulation of HD and LT unexpectedly not by activating platelets, but by inducing spontaneous clotting originating from the particles itself. Therefore, the expected EDMP-mediated platelet activation via membrane P-selectin seems to play a secondary role in coagulation stimulation, which is likely due to a lower P-selectin exposure present on in vitro generated EDMP, which seem to be insufficient to activate platelets in a way PDMP do.

Furthermore, we observed a concentration-dependent mode of action for both PDMP and EDMP as both particles revealed a stronger effect in higher concentrations (10 k or 15 k MP/μl blood). For EDMP, the concentration effect mainly accelerated spontaneous clotting in HD (< 5 min) and LT (< 3 min) conditions as the process itself was not concentration-dependent. Already low EDMP concentrations (1 k MP/μl blood) led to spontaneous fibrin formation. An underlying mechanism by which EDMP cause spontaneous clotting could be based on the presence of tissue factor (TF) on EDMP, which has been shown to be released after targeted stimulation from human endothelial cells in vitro [41–43]. TF in turn mediates a coagulation initiation via the coagulation factor VII/TF-dependent extrinsic pathway and likely results in spontaneous fibrin formation [44, 45]. The earlier formation of spontaneous fibrin clots following EDMP administration in LT in comparison to HD potentially resulted from the combination of both, the TF-bearing EDMP and the acidosis-related increased basal platelet activation under LT conditions.

As recently published by our group, there is a positive correlation between injury severity and MP concentration after trauma likely reaching high quantities inducing procoagulant effects [25]. Our in vitro experimental setup indicates that under LT conditions MP promote procoagulant function by different pathways based on their cellular origin. Therefore, at first sight, our data seems to be contrary to recent clinical observations of platelet dysfunction in TIC-related patients showing reduced platelet activity levels after trauma [16, 17]. These clinical data reflect the final common path of the multicausal pathophysiology of TIC resulting in the hypocoagulative state after trauma. The results from our experimental model should only be carefully extrapolated to a clinical picture but describes the procoagulant potential. It remains to be conclusively clarified and requires further investigation whether and how MP are involved in the pathophysiology of TIC after major trauma in vivo and whether those high quantities as used in this study can, in fact, be physiologically achieved in a clinical setting.

## Conclusion

The present study revealed a procoagulant role of PDMP and EDMP under circumstances of a dilutional coagulopathy as it occurs after major traumatic injury. Particularly, high concentrations of both MP types showed effects on the coagulation process. A divergent mechanism of action indicate that these MP subtypes have diverse function in stimulating coagulation under the investigated conditions of HD and LT. Likely due to the platelet-mediated activation (increased P-selectin expression) and the presence of PS lipids on PDMP membranes, clotting factor assembly and thus clot formation could be promoted on PDMP surfaces. In association with high EDMP quantities, an improved coagulation with formation of spontaneous fibrin clots was determined, which could presumably be induced via the TF/FVIIa-dependent extrinsic pathway of coagulation.

### Limitations

It would have been desirable to perform the analysis with isolated MP subtypes from real trauma patients. But since high MP quantities would have been required for this study and the extraction capacity from patient’s blood is limited, we relied on in vitro synthesized MP. There is a limitation in interpreting the results as in vitro LPS-stimulated MP might have a higher procoagulant activity compared to in vivo MP [46]. However, during the validation process of our assays, we compared the activity of in vitro generated MP to that of in vivo MP and did not see any difference.

Additionally, we are aware of the fact that the current in vitro study reflects the pathophysiological mechanism present in trauma patients only in part. For this reason, findings should cautiously be transferred to a clinical setting.

## Data Availability

All data that are relevant for the study are included in this published article. Further datasets analyzed during the current study are available from the corresponding author on reasonable request.

## Abbreviations

ATC: Acute traumatic coagulopathy
C_S_: Clot size
CMMC: Cologne-Merheim Medical Centre
EDMP: Endothelial-derived microparticles
D: Clot density
.: Endothelial-derived microparticles
HD: Haemodilution
IC: Iatrogenic coagulopathy
ITM: Institute of Transfusion Medicine
LPS: Lipopolysaccharide
LT: Lethal triad
MP: Microparticles
PDMP: Platelet-derived microparticles
PPP: platelet-poor plasma
PS: Phosphatidylserine
PSGL-1: P-Selectin gylcoprotein ligand-1
TD: Thrombodynamics
TIC: Trauma-induced coagulopathy
Tlag: lag-time
TF: Tissue factor
Tsp: Time of spontaneous clot formation
V: Rate of clot growth
Vi: Initial rate of clot growth
WB: Whole blood

## References

[1] Krug EG, Sharma GK, Lozano R. The global burden of injuries. Am J Public Health. 2000;90:523–6. 10.2105/ajph.90.4.523 PMC1446200 10754963

[2] Mathers CD, Loncar D (2006). Projections of global mortality and burden of disease from 2002 to 2030. PLoS Med.

[3] Kushimoto S, Kudo D, Kawazoe Y. Acute traumatic coagulopathy and trauma-induced coagulopathy: an overview. J Intensive Care. 2017;5:6. 10.1186/s40560-016-0196-6 PMC8600738 34798701

[4] Holcomb JB, Tilley BC, Baraniuk S, Fox EE, Wade CE, Podbielski JM (2015). Transfusion of plasma, platelets, and red blood cells in a 1:1:1 vs a 1:1:2 ratio and mortality in patients with severe trauma: the PROPPR randomized clinical trial. JAMA - J Am Med Assoc.

[5] Sauaia A, Moore FA, Moore EE, Moser KS, Brennan R, Read RA (1995). Epidemiology of trauma deaths: a reassessment. J Trauma.

[6] Brohi K, Singh J, Heron M, Coats T (2003). Acute traumatic coagulopathy. J Trauma.

[7] Maegele M, Lefering R, Yucel N, Tjardes T, Rixen D, Paffrath T (2007). Early coagulopathy in multiple injury: an analysis from the German trauma registry on 8724 patients. Injury..

[8] MacLeod JBA, Lynn M, McKenney MG, Cohn SM, Murtha M (2003). Early coagulopathy predicts mortality in trauma. J Trauma.

[9] Caspers M, Maegele M, Fröhlich M. Current strategies for hemostatic control in acute trauma hemorrhage and trauma-induced coagulopathy. Expert Rev Hematol. 2018;11(12):987–95. 10.1080/17474086.2018.1548929 30433835

[10] Meledeo MA, Herzig MC, Bynum JA, Wu X, Ramasubramanian AK, Darlington DN, et al. Acute traumatic coagulopathy: the elephant in a room of blind scientists. J Trauma Acute Care Surg. 2017;2:33–40. 10.1097/TA.0000000000001431 28333829

[11] Stensballe J, Henriksen HH, Johansson PI. Early haemorrhage control and management of trauma-induced coagulopathy: The importance of goaldirected therapy. Curr Opin Crit Care. 2017;23:503–10. 10.1097/MCC.0000000000000466 29059118

[12] Maegele M, Schöchl H, Cohen MJ. An update on the coagulopathy of trauma. Shock. 2014. 10.1097/SHK.0000000000000088 24192549

[13] Gando S, Sawamura A, Hayakawa M (2011). Trauma, shock, and disseminated intravascular coagulation: lessons from the classical literature. Ann Surg.

[14] Noel P, Cashen S, Patel B (2013). Trauma-induced coagulopathy: from biology to therapy. Semin Hematol.

[15] Meng ZH, Wolberg AS, Monroe DM, Hoffman M. The effect of temperature and pH on the activity of factor VIIa: implications for the efficacy of high-dose factor VIIa in hypothermic and acidotic patients. J Trauma. 2003;55:886–9. 10.1097/01.TA.0000066184.20808.A5 14608161

[16] Wohlauer MV, Moore EE, Thomas S, Sauaia A, Evans E, Harr J (2012). Early platelet dysfunction: an unrecognized role in the acute coagulopathy of trauma. J Am Coll Surg.

[17] Ramsey MT, Fabian TC, Shahan CP, Sharpe JP, Mabry SE, Weinberg JA (2016). A prospective study of platelet function in trauma patients. J Trauma Acute Care Surg.

[18] Park MS, Xue A, Spears GM, Halling TM, Ferrara MJ, Kuntz MM (2015). Thrombin generation and procoagulant microparticle profiles after acute trauma. J Trauma Acute Care Surg.

[19] Curry N, Raja A, Beavis J, Stanworth S, Harrison P. Levels of procoagulant microvesicles are elevated after traumatic injury and platelet microvesicles are negatively correlated with mortality. J Extracell. vesicles. 2014;3:25625. 10.3402/jev.v3.25625 PMC4216813 26077419

[20] Windeløv NA, Johansson PI, Sørensen AM, Perner A, Wanscher M, Larsen CF (2014). Low level of procoagulant platelet microparticles is associated with impaired coagulation and transfusion requirements in trauma patients. J Trauma Acute Care Surg.

[21] Balvers K, Curry N, Kleinveld DJB, Böing AN, Nieuwland R, Goslings JC (2015). Endogenous microparticles drive the Proinflammatory host immune response in severely injured trauma patients. Shock..

[22] Xie RF, Hu P, Li W, Ren YN, Yang J, Yang YM (2014). The effect of platelet-derived microparticles in stored apheresis platelet concentrates on polymorphonuclear leucocyte respiratory burst. Vox Sang.

[23] Gruen RL, Brohi K, Schreiber M, Balogh ZJ, Pitt V, Narayan M (2012). Haemorrhage control in severely injured patients. Lancet..

[24] Matijevic N, Wang YWW, Wade CE, Holcomb JB, Cotton BA, Schreiber MA, et al. Cellular microparticle and thrombogram phenotypes in the prospective observational Multicenter major trauma transfusion (PROMMTT) study: correlation with coagulopathy. Thromb Res. 2014;134(3):652–8. 10.1016/j.thromres.2014.07.023 PMC4160305 25086657

[25] Fröhlich M, Schäfer N, Caspers M, Böhm JK, Stürmer EK, Bouillon B, et al. Temporal phenotyping of circulating microparticles after trauma: a prospective cohort study. Scand J Trauma Resusc Emerg Med. 2018;27;26(1):33. 10.1186/s13049-018-0499-9 PMC5921785 29703240

[26] Wolberg AS, Monroe DM, Roberts HR, Hoffman MR (1999). Tissue factor de-encryption: ionophore treatment induces changes in tissue factor activity by phosphatidylserine-dependent and -independent mechanisms. Blood Coagul Fibrinolysis.

[27] Khan MMH, Hattori T, Niewiarowski S, Edmunds LH, Colman RW (2006). Truncated and microparticle-free soluble tissue factor bound to peripheral monocytes preferentially activate factor VII. Thromb Haemost.

[28] Schäfer N, Driessen A, Bauerfeind U, Fröhlich M, Ofir J, Stürmer EK (2016). *In vitro* effects of different sources of fibrinogen supplementation on clot initiation and stability in a model of dilutional coagulopathy. Transfus Med.

[29] Caspers M, Schäfer N, Fröhlich M, Bauerfeind U, Bouillon B, Mutschler M, et al. How do external factors contribute to the hypocoagulative state in trauma-induced coagulopathy? - in vitro analysis of the lethal triad in trauma. Scand J Trauma Resusc Emerg Med. 2018;26:66. 10.1186/s13049-018-0536-8 PMC6094881 30111342

[30] Ruf APH (1995). Flow cytometric detection of activated platelets: comparison of determining shape change, fibrinogen binding, and P-selectin expression. Semin Thromb Hemost.

[31] Caspers M, Schäfer N, Fröhlich M, Bouillon B, Mutschler M, Bauerfeind U, et al. Microparticles profiling in trauma patients: high level of microparticles induce activation of platelets in vitro. Eur J Trauma Emerg Surg. 2020;46(1):43–51. 10.1007/s00068-019-01111-7 30864053

[32] Frenette PS, Denis CV, Weiss L, Jurk K, Subbarao S, Kehrel B (2000). P-Selectin glycoprotein ligand 1 (Psgl-1) is expressed on platelets and can mediate platelet–endothelial interactions in vivo. J Exp Med.

[33] Coenen DM, Mastenbroek TG, Cosemans JMEM (2017). Platelet interaction with activated endothelium: mechanistic insights from microfluidics. Blood..

[34] Galbusera M, Buelli S, Gastoldi S, Macconi D, Angioletti S, Testa C (2005). Activation of porcine endothelium in response to xenogeneic serum causes thrombosis independently of platelet activation. Xenotransplantation..

[35] Martins PDC, García-Vallejo JJ, Van Thienen JV, Fernandez-Borja M, Van Gils JM, Beckers C (2007). P-selectin glycoprotein ligand-1 is expressed on endothelial cells and mediates monocyte adhesion to activated endothelium. Arterioscler Thromb Vasc Biol.

[36] Kuravi SJ, Yates CM, Foster M, Harrison P, Hazeldine J, Hampson P, et al. Changes in the pattern of plasma extracellular vesicles after severe trauma. PLoS One. 2017;24;12(8):e0183640. 10.1371/journal.pone.0183640 PMC5570308 28837705

[37] Reddy EC, Wang H, Christensen H, McMillan-Ward E, Israels SJ, Bang KWA, et al. Analysis of procoagulant phosphatidylserine-exposing platelets by imaging flow cytometry. Res Pract Thromb Haemost. 2018;2:736–50. 10.1002/rth2.12144 PMC6178738 30349893

[38] Zhao L, Bi Y, Kou J, Shi J, Piao D (2016). Phosphatidylserine exposing-platelets and microparticles promote procoagulant activity in colon cancer patients. J Exp Clin Cancer Res.

[39] Zhang Y, Meng H, Ma R, He Z, Wu X, Cao M (2016). Circulating microparticles, blood cells, and endothelium induce procoagulant activity in sepsis through phosphatidylserine exposure. Shock..

[40] Lipets EN, Antonova OA, Shustova ON, Losenkova KV, Mazurov AV, Ataullakhanov FI. Use of Thrombodynamics for revealing the participation of platelet, erythrocyte, endothelial, and monocyte microparticles in coagulation activation and propagation. PLoS One. 2020;15(5):e0227932. 10.1371/journal.pone.0227932 PMC7259734 32469873

[41] Khaspekova SG, Antonova OA, Shustova ON, Yakushkin VV, Golubeva NV, Titaeva EV (2016). Activity of tissue factor in microparticles produced in vitro by endothelial cells, monocytes, granulocytes, and platelets. Biochem..

[42] Kagawa H, Komiyama Y, Nakamura S, Miyake T, Miyazaki Y, Hamamoto K (1998). Expression of functional tissue factor on small vesicles of lipopolysaccharide-stimulated human vascular endothelial cells. Thromb Res.

[43] Nomura S, Shouzu A, Omoto S, Nishikawa M, Iwasaka T, Fukuhara S (2004). Activated platelet and oxidized LDL induce endothelial membrane vesiculation: clinical significance of endothelial cell-derived microparticles in patients with type 2 diabetes. Clin Appl Thromb.

[44] Cimmino G, Cirillo P. Tissue factor: Newer concepts in thrombosis and its role beyond thrombosis and hemostasis. Cardiovasc Diagn Ther. 2018;8(5):581–93. 10.21037/cdt.2018.10.14 PMC6232348 30498683

[45] MacKman N. The role of tissue factor and factor VIIa in hemostasis. Anesth Analg. 2009;108(5):1447–52. 10.1213/ane.0b013e31819bceb1 PMC2838713 19372318

[46] Bernimoulin M, Waters EK, Foy M, Steele BM, Sullivan M, Falet H, et al. Differential stimulation of monocytic cells results in distinct populations of microparticles. J Thromb Haemost. 2009;7(6):1019–28. 10.1111/j.1538-7836.2009.03434.x PMC3242443 19548909

